# Spatial and Temporal Dynamics of a Mortality Event among Central African Great Apes

**DOI:** 10.1371/journal.pone.0154505

**Published:** 2016-05-18

**Authors:** Kenneth N. Cameron, Patricia Reed, David B. Morgan, Alain I. Ondzié, Crickette M. Sanz, Hjalmar S. Kühl, Sarah H. Olson, Eric Leroy, William B. Karesh, Roger Mundry

**Affiliations:** 1 Wildlife Health & Health Policy Program, Wildlife Conservation Society, Bronx, New York, United States of America; 2 Global Conservation Program, Wildlife Conservation Society, Bronx, New York, United States of America; 3 Lester E. Fisher Center for the Study and Conservation of Apes, Lincoln Park Zoo, Chicago, Illinois, United States of America; 4 Department of Anthropology, Washington University, Saint Louis, Missouri, United States of America; 5 Max Planck Institute for Evolutionary Anthropology, Department of Primatology, Leipzig, Germany; 6 German Centre for Integrative Biodiversity Research (iDiv) Halle-Leipzig-Jena, Leipzig, Germany; 7 Center for Sustainability and the Global Environment (SAGE), University of Wisconsin, Madison, Wisconsin, United States of America; 8 Centre International de Recherches Médicales de Franceville, Institut de Recherches pour le Développement, Franceville, Gabon; Division of Clinical Research, UNITED STATES

## Abstract

In 2006–2007 we observed an unusual mortality event among apes in northern Republic of Congo that, although not diagnostically confirmed, we believe to have been a disease outbreak. In 2007–2011 we conducted ape nest surveys in the region, recording 11,835 *G*. *g*. *gorilla* nests (2,262 groups) and 5,548 *P*. *t*. *troglodytes* nests (2,139 groups). We developed a statistical model to determine likely points of origin of the outbreak to help identify variables associated with disease emergence and spread. We modeled disease spread across the study area, using suitable habitat conditions for apes as proxy for local ape densities. Infectious status outputs from that spread model were then used alongside vegetation, temperature, precipitation and human impact factors as explanatory variables in a Generalized Linear Model framework to explain observed 2007–2011 ape nest trends in the region. The best models predicted emergence in the western region of Odzala-Kokoua National Park and north of the last confirmed Ebola virus disease epizootics. Roads were consistently associated with attenuation of modeled virus spread. As disease is amongst the leading threats to great apes, gaining a better understanding of disease transmission dynamics in these species is imperative. Identifying ecological drivers underpinning a disease emergence event and transmission dynamics in apes is critical to creating better predictive models to guide wildlife management, develop potential protective measures for wildlife and to reduce potential zoonotic transmission to humans. The results of our model represent an important step in understanding variables related to great ape disease ecology in Central Africa.

## Introduction

The Congo basin of Central Africa is home to roughly 80% of western lowland gorillas (*Gorilla gorilla gorilla*) and central chimpanzees (*Pan troglodytes troglodytes*), categorized as “critically endangered” and “endangered”, respectively. Major threats to these species include hunting, habitat loss and disease [1]. The Congo Basin is also home to 300 million people, many of who rely heavily on coexistence with, and consumption of, wildlife. Zoonotic emerging infectious diseases (EIDs) have increased significantly in recent decades, with roughly 70% originating in wildlife [2]. Disappearing ecological barriers, caused by expanding exploitation of natural resources and the resultant increase in human-wildlife overlap that typify most remaining forests are thought to be at the origin of many wildlife-to-human disease spillover events. Yet humans are not the only anthropoid species to be affected by these emerging diseases. Over the past roughly 20 years numerous mortality events have occurred amongst great apes in the greater Gabon and Republic of Congo (Congo) region of Central Africa, typically in regions of high ape density [3,4]. The cause(s) of these mass mortality events have usually not been definitively determined, but the broad extent, seemingly rapid occurrence and apparent density-dependent nature of the die-offs strongly suggest a highly pathogenic infectious disease. Ebola virus disease (EVD), in particular, has been proposed as a likely cause of many, if not the majority, of these die-offs [4,5,6,7].

Other significant great ape population declines not associated with known mortality have been documented in this region. A large mammal survey in Congo’s Odzala-Kokoua National Park (OKNP) found a nearly 50% decline in *G*. *g*. *gorilla* abundance estimates from 2005 to 2012 and proposed infectious disease as the likely cause [2,4]. In 2012 the densities in the park had dropped to 1.62 gorillas/km2 from 3.03 gorillas/km2 in 2005.

**Fig 1:**
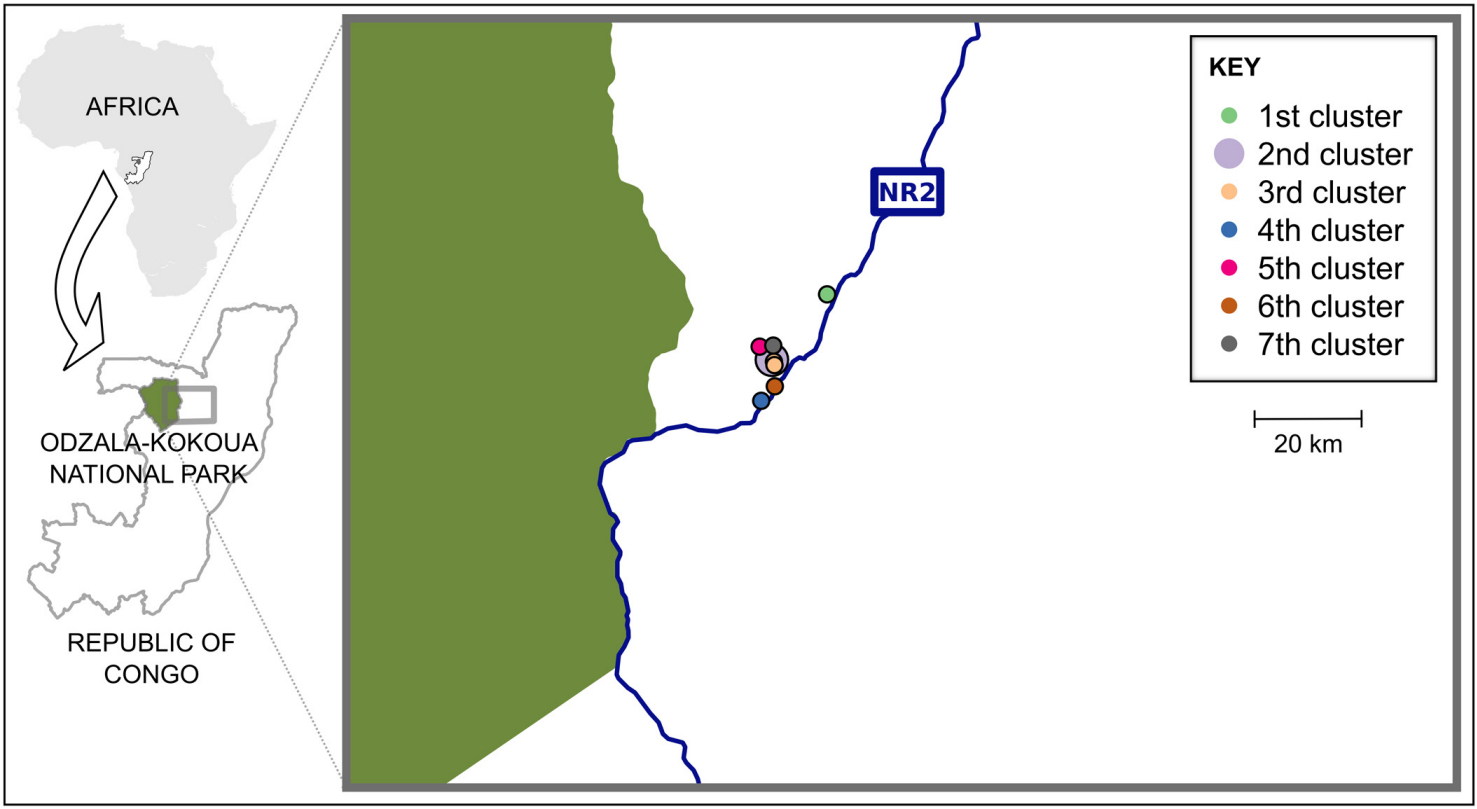
Map of the of great ape carcasses discovered in 2006–2007 in the Odzala region of Congo. Circles indicate locations of great ape carcasses. Carcass clusters are differentiated by color (see in-figure legend). OKNP is indicated in green. The blue line indicates National Route 2 (NR2). The area shown is enlarged in Figs 3 and 4.

In late 2006 through early 2007, we observed an unusual mortality event among great ape populations in the Sangha Department of northern Congo. Over a period of three months, nine great ape carcasses (one *P*. *t*. *troglodytes*, eight *G*. *g*. *gorilla*) were discovered in seven “clusters” of one to three carcasses in the vicinity of the north-south road National Route 2 (NR2) (Fig 1). On November 2, 2006 a field team conducting line transect surveys for estimation of large mammal abundance discovered the moderately decomposed carcass of a single juvenile female chimpanzee (first cluster). A veterinary team collected several dried skin samples in RNAlater^®^ (RNAlater^®^ Stabilization Solution, Ambion^™^) for possible diagnostic testing. On January 14, 2007 two hunters reported five gorilla carcasses near Libonga village along NR2. Those carcasses had been discovered between December 20 and 24, 2006. One hunter had discovered the fresh carcass of a juvenile gorilla, the moderately decomposed carcass of an adult female and the fresh carcass of a juvenile, all within 3–5 m of each other (second cluster). During the same 4-day period the second hunter had independently discovered two additional gorilla carcasses (third cluster); an adult female and an adult male, both largely decomposed, within 400–500 m of each other. A veterinary team verified the presence of four of the carcasses, but only hair and soil staining from the bodily fluids remained of the fifth (juvenile female). Samples (skull and long bones) were collected from the four carcasses. On January 22, 2007 villagers discovered the carcass of an adult male gorilla (fourth cluster) in the Louamé River. A veterinary team collected soft tissue samples (moist skin & skeletal muscle) in RNAlater^®^. On January 31, 2007 villagers discovered the carcass of an unidentified gorilla (fifth cluster) near the Miteba River. A veterinary team collected bones.

Seeking to find any additional carcasses to better characterize the mortality event, we conducted multiple directed reconnaissance walk surveys (recces) in the vicinity of the carcass clusters. During the first two recces, centered near the second cluster, teams discovered two additional gorilla carcasses; an adult female (sixth cluster) on February and an adult male (seventh cluster) on February 23, 2007 both in advanced state of decomposition (only bones remaining) (Fig 1). Teams collected skulls from both. Pending diagnostic testing, we stored tissue samples in RNAlater^®^ at -4°C and bones at -20°C.

Samples from five carcasses (from second, third and fourth clusters) were sent in late January 2007 to the Centre International de Recherches Médicales de Franceville (CIRMF), a regional reference laboratory in Gabon, for pathogen testing. CIRMF considered only the moist soft tissue sample from the third cluster to be of adequate quality for diagnostic testing. That sample tested negative for ebolaviruses and Marburg virus by reverse transcriptase polymerase chain reaction (RT-PCR) and antigen capture assays in early February 2007. No additional pathogen testing was conducted. We detected no evidence of hunting-related trauma on any of the carcasses. Recce data, market monitoring, hunter interviews and the lack of physical evidence revealed no indication of poaching as a cause of the mortality. Despite the negative test results, and based on the spatial and temporal patterns of mortality, we presumed this unusual mortality event to be the result of a disease outbreak.

In the present study we sought to develop a statistical model to determine likely points of origin of this presumed disease outbreak. We first modeled disease spread across the study area. As ape density data from this region is scarce, we used published suitable environmental conditions for great apes habitat model [8] as a proxy for local *G*. *g*. *gorilla* and *P*. *t*. *troglodytes* densities. Infectious status outputs from spread simulations were then used alongside environmental parameters as explanatory variables in a Generalized Linear Model (GLM) framework to model observed ape nest trends recorded from 2005–2011 along directed reconnaissance walk surveys (recces). Our overall goal was to help identify aspects of landscape matrix and connectivity that correspond to patterns of disease emergence, spread and termination in great ape populations.

## Materials and Methods

### Study area

The study area was centered along NR2 in the Sangha Department of the Congo (0.35°–1.37° N; 15.00°–15.99° E). The specific areas that we identified as priority for this survey were within the Ngombé Forestry Management Unit east of OKNP, a region of suspected large-scale great ape mortality associated with EVD outbreaks. The climate in northern Congo can be described as transitional between the Congo-equatorial and sub-equatorial climatic zones. Rainfall is bimodal, with a main rainy season from August through November and a short rainy season in May.

### Guided reconnaissance walk surveys

Between 2005 and 2011 we conducted guided reconnaissance walk surveys (recces) [9] at, or in the vicinity of, locations where great ape carcasses had been found near villages along NR2, east of OKNP. Each survey zone consisted of four segments that radiated 10 km from a central point [10]. Each segment was comprised of three lines. Team leaders navigated between segment start points and end points using handheld GPS units (Garmin 60CSx). A compass bearer walked in front of the transect observer to guide the path of travel along the pre-determined compass bearing. Team leaders recorded ape signs, including nests, direct observations, vocalizations, feeding remains, tracks and feces. To estimate human presence as proxy for hunting pressure, team leaders also recorded human sign, including camps, cartridges, machete marks, footprints, gunshots, vocalizations, etc. We conducted in sixteen different zones, first nearest the carcass clusters, then progressively farther away. We repeated eleven of the recces over time, at random intervals and as resources permitted, in order to establish temporal trends in great ape populations. In 2007 we also repeated an additional 50 km U-shaped recce on the northeast side of the Mambili River within OKNP, which was first conducted in response to ape mortality and two confirmed *Zaïre ebolavirus*-positive primate carcasses in 2005 [11].

Over subsequent months, as no additional ape carcasses were discovered, recces were continued to try to determine ape population trends over time. We estimated ape nest ages using definitions and methods based on published methods [12].

### Data processing

Any nests that were more than 1,000 m from the closest recce segment of the respective zone were removed from the analysis. There were few of these outlying nests and these were likely due to observer transcription or GPS errors. We split recce segments into fragments of equal length, each being as near as possible to 1,000 m. We then assigned each nest to the fragment and zone to which it was closest. For each fragment we determined nests and nest group counts separately for *P*. *t*. *troglodytes* and *G*. *g*. *gorilla*, as well as per recce mission, which we then used as response variables in the analysis. We also assigned the middle of the survey start and finish dates as the observation date of the fragment and the center point of the fragment as its location.

Extraction of environmental and human impact covariates

To account for varying environmental conditions, degrees of human influence, etc., we extracted a total of 26 variables at various resolutions (see S1 Table for details). With the exception of habitat type, each recce fragment was assigned the value of the closest grid cell in the respective covariates map. For habitat type, we determined the relative frequency distribution of the various habitat types within a circle of 1 km radius around each fragment's center point. Since only three habitat types were common in the study area, habitat type was expressed using two variables indicating percentage cover with closed to open canopy (comprised of broadleaved evergreen (>15%) or semi-deciduous forest (>5m)), subsequently referred to as ‘Forest’, and mosaic vegetation (comprised of grassland/shrubland/forest (50–70%)/cropland (20–50%)), subsequently referred to as ‘Mosaic’.

We subjected all environmental and human impact covariates to a Factor Analysis (FA) with varimax rotation to reduce the number of predictors and avoid redundancy among them. This was justified by partly large correlations between the covariates. The FA revealed six factors with Eigenvalues in excess of one, together explaining 87.4% of the total variance (S2 Table). Prior to running the FA, we transformed variables, where required, to achieve approximately symmetrical distributions based on visual assessment (S1 Table).

### Modeling density-dependent infectivity

The disease spread model was based on a 5 km x 5 km grid ranging from 0.521° S to 2.230° N and 14.109° E to 17.192° E (cells northeast of the Sangha River were not considered). We systematically modeled the disease originating at each of the 4,050 cells of the grid. A stochastic process based on the suitable environmental conditions (SEC) for great apes [13] of each cell governed disease spread from the cell of origin. Suitability values were divided by their maximum in the map such that their maximum equaled one (minimum equal to 0.0074). At each time step of the simulation, we first determined the cell’s neighboring infectious cells, defined as the four cells sharing sides with an infected cell. Infected cells were infectious for two time steps. In the first time step an infected cell's risk of infecting a neighboring cell was set to one. In the second time step this risk was reduced to a quarter (see below), and after two time steps cells were not considered infectious. The probability that a neighboring cell became infected was set to the product of its habitat suitability value and the infected cell’s infectious risk. When an uninfected cell had more than one infectious neighboring cell, its probability of becoming infected was determined as one minus the product of its probabilities of not becoming infected by its different neighboring cells being infectious. To summarize, the risk of a given cell becoming infected was $R_{i}=1-\prod_{k=1}^{n}\left(1-SEC_{i}*R_{k}\right)$, where *R*_*i*_ and *SEC*_*i*_ are the risk of becoming infected and environmental suitability (SEC), respectively, of the *i*^th^ cell, *R*_*k*_ is the infection probability of the *k*^th^ neighboring cell (being 1 at time step 1 after its infection, 0.25 at the next time step and 0 thereafter), and *n* is the number of infectious neighbors of the *i*^th^ cell (at most four).

A random draw from a binomial distribution (1: infected; 0: uninfected), according to the cell’s probability of becoming infected, determined whether an infection actually took place. A simulation stopped when either all cells on the map were infected or the spread of the disease stopped since no more infectious cells were available. During each simulation, any given cell could become infected at most once.

A time step was considered to last 30 days, which was our *a priori* approximation of the speed at which the disease progressed across 5 km (the size of the grid cells) and our model time step. We assumed that once a population disappeared after an outbreak in a cell, the risk of infection approached zero after approximately seven days. Therefore, we set the infectious risk to a quarter of its original value in the second time step after becoming infected and to zero thereafter.

Following each simulation, we retrieved infection information for each grid cell and, if infected, at which time step it became infected. We assume that the observed 2006–2007 outbreak actually began between 1 January 2004 and mid-May 2005. We based this assumption on considerations regarding the size of the area and the assumed speed of spread of a disease like Ebola. It is also worth noting that if outbreaks in the region within this period do not explain the change of great ape abundance we found along the recces, our models would not reveal a positive result (i.e. the null model of no impact of an outbreak would be supported as the best result). Basing this assumption on the documented history of EVD epizootics west of our study region (implemented with an increment of 30 days), we eventually came up with different scenarios for when and where the outbreak originated and to what spatio-temporal patterns of cells being affected or not these scenarios could have led. To deal with stochasticity in the simulation we conducted 100 simulations per cell of origin.

### Statistical modeling of the impact of disease outbreaks and spreads

The basic analysis was a Generalized Linear Model (GLM) [14] with negative binomial error structure and log link function. As predictors in this model we included the six environmental and human impact factors (see above), infection status and autocorrelation (see below). Response variables were *P*. *t*. *troglodytes* or *G*. *g*. *gorilla* total nests or nest group counts, analyzed one at a time. In the model we included all two-way interactions between infection status, on the one hand, and the environmental and human impact factors, on the other hand, because it seems likely that the effects of environmental and human impact covariates on ape abundance could dramatically change as a consequence of the outbreak (e.g., a positive impact of an environmental gradient on ape abundance can only be effective and detected if apes are present in the area). Infection status was positive if, at the time of the survey, the cell that contained the fragment had been affected by the simulated outbreak. Otherwise, infection status was negative. When none of the fragments was affected by a simulated outbreak, we discarded the infection status variable and modeled the response solely as a function of the six factors representing environmental gradients and human impact.

We explicitly included spatio-temporal autocorrelation into the model because the data analyzed were likely to show this property beyond what is explained by the included predictors. Such autocorrelation would lead to non-independent residuals (i.e., residuals derived for fragments sampled closer to one another in time and space being more similar than those from fragments sampled further apart), reducing the validity of the statistical model. We addressed this issue by first running the full model as described above and extracting its residuals. For each data point, we then averaged the residuals from its spatio-temporal neighborhood and included the derived averages as an additional variable (‘autocorrelation term’) into the model. The spatio-temporal neighborhood was defined as those fragments sampled at a distance of at most 10 km and within 30 days. Finally, to account for varying lengths of fragments, we included it (log-transformed) as an offset term into the model. Prior to running the model, the environmental and human impact covariates and the autocorrelation term were z-transformed to a mean of zero and a standard deviation of one. Since occasionally the GLM with negative binomial error structure did not converge we first ran a GLM with Poisson error structure and used the derived estimated coefficients as starting values for the estimation of the GLM with negative binomial error structure. We extracted the Akaike Information Criterion (AIC) to measure the fit of the final model [15] and when the model did not converge we set the AIC to missing.

### Determination of likely points of disease origin

Overall, we ran 4,050 (cells of possible origin) times 100 (simulations) times 17 (outbreak dates) = 6,885,000 models for each of the four response variables (*G*. *g*. *gorilla* and *P*. *t*. *troglodytes* nest and nest group counts). The total number of models that converged was 6,711,072 for *G*. *g*. *gorilla* nests, 6,711,214 for *P*. *t*. *troglodytes* nests, 6,715,969 for *G*. *g*. *gorilla* nest groups and 6,717,401 for *P*. *t*. *troglodytes* nest groups. Akaike weights were used to summarize the models that converged. Separately for each response variable, we then determined and inspected the 95% best model confidence set.

### Implementation

With one exception, the analyses including the simulation were run in R [16]. GLMs were run using the function glm.nb from the R package MASS [17]. The factor analysis was conducted in SPSS for Windows version 15.0.1.

## Results

### Ape population monitoring

**Fig 2:**
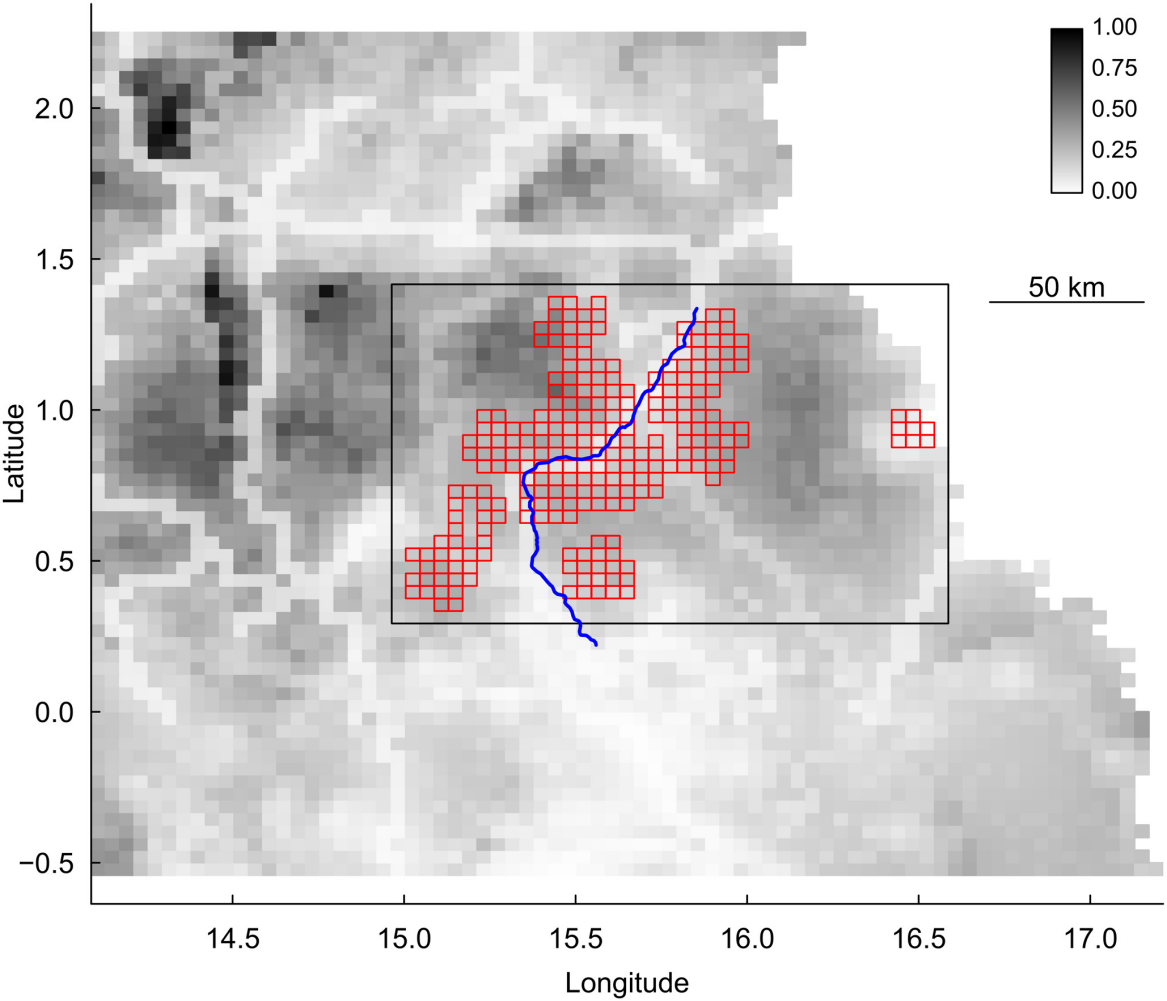
Map of the of the survey design in the Odzala region in Congo. The grey shading of the background reflects habitat suitability for great apes (with darker tones indicating more suitable habitat). The blue line depicts National Route 2, and cells framed in red are those surveyed as part of the project. The black rectangle in the center indicates area shown enlarged in Figs 3 and 4.

In response to the 2006–2007 ape mortality event, we conducted additional recces to increase geographic coverage in order to document the extent and progression of the die-off and to sample carcasses for diagnostic testing. From March 2007 through December 2011 we conducted 4,355 km of recces in the OKNP/peri-OKNP region (Fig 2). We recorded 11,835 *G*. *g*. *gorilla* nests in 2,262 groups and 5,548 *P*. *t*. *troglodytes* nests in 2,139 groups, for an average of four ape nests and one nest group per kilometer surveyed.

**Fig 3:**
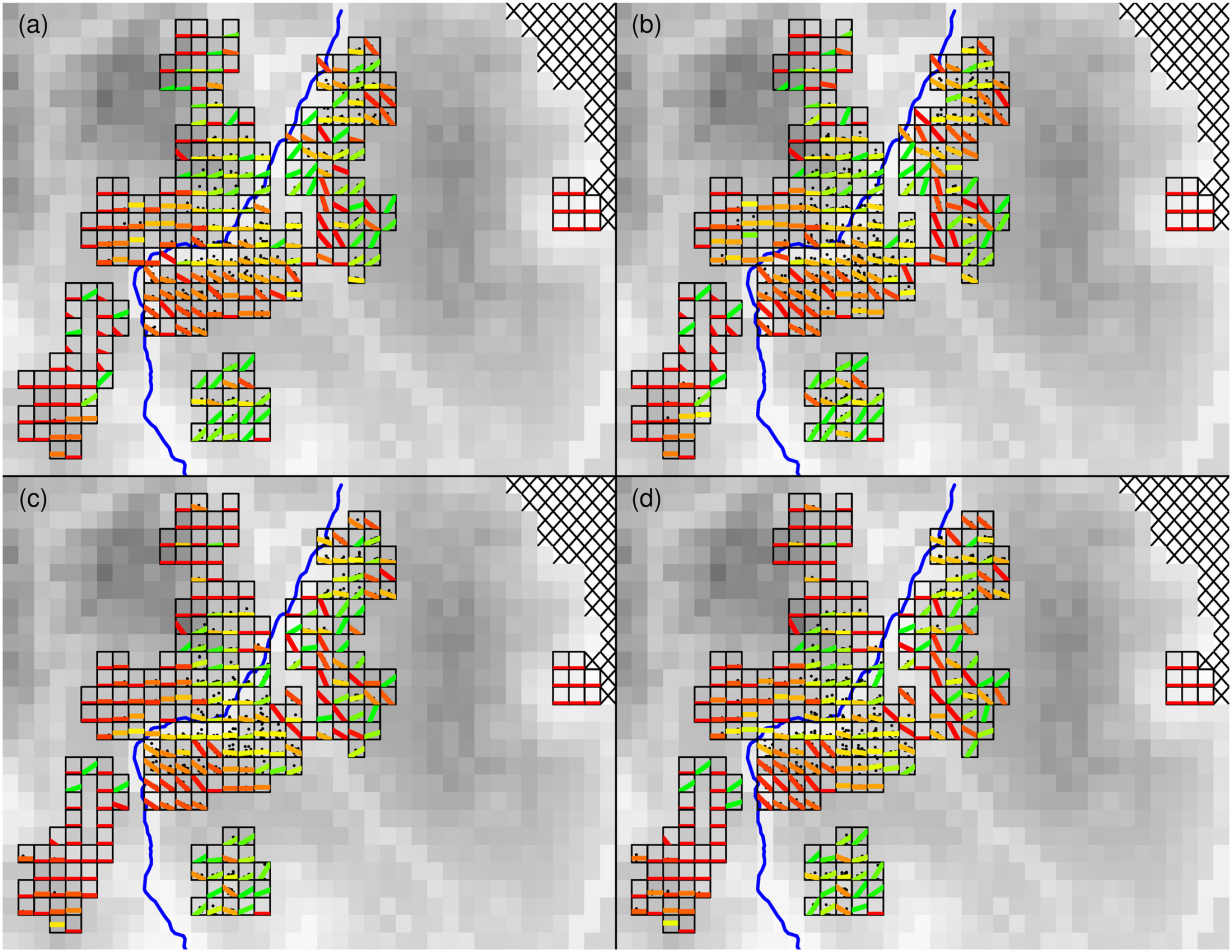
Spatio-temporal patterns of ape nest abundance in the study area separately for *G*. *g*. *gorilla* nests and nest groups (a and b, respectively), and *P*. *t*. *troglodytes* nests and nest groups (c and d, respectively). Within each cell, the numbers of nests or nest groups (square-root transformed) is plotted against the time when the surveys took place (axes are at the same scale within panels (a, b, c, or d), but not across them). For illustrative purposes, in cells where there were at least two surveys separated by at least 30 days, we show least squares regression lines (red, yellow and green lines within cells) between number of nests or nest groups and time. In the other cells we show the average number of nests as a horizontal line. Regression lines were colored according to the correlation coefficient (rho) between number of nests or nest groups and time, with colors grading from red (rho = -1) over yellow (rho = 0) to green (rho = 1). Horizontal lines were colored grading from red (no nests or nest groups) over yellow (half the maximum number of nests or nest groups) to green (maximum number of nests or nest groups). The grey shading of the background reflects habitat suitability for great apes (with darker tones indicating more suitable habitat). Note that to the west of the road (NR2; blue line) most cells had either low or clearly decreasing ape nest densities, whereas to the east of the road neighboring cells showed strikingly differing patterns. In each panel, the depicted area equals that within the rectangle shown in Fig 2.

The vast majority (89.1%) of the total of 26,855,656 simulations (for the four responses, namely *G*. *g*. *gorilla* and *P*. *t*. *troglodytes* nest and nest group counts) did not affect any of the cells where our recces took place (range across the four models was 89.1%-89.2%) (Fig 3). Where numbers of ape nests and nest groups were increasing west of NR2, the increases were usually shallow (Fig 3). East of the road the patterns were inconsistent, with some cells indicating clear increases and other cells clear decreases being partly interspersed amongst one another. However, in the easternmost cells we did not find any ape nests. Furthermore, below the southeast bend of the road there was a cluster of neighboring cells that showed a clear decline over the study period.

### Modeling the origin of disease outbreak

**Fig 4:**
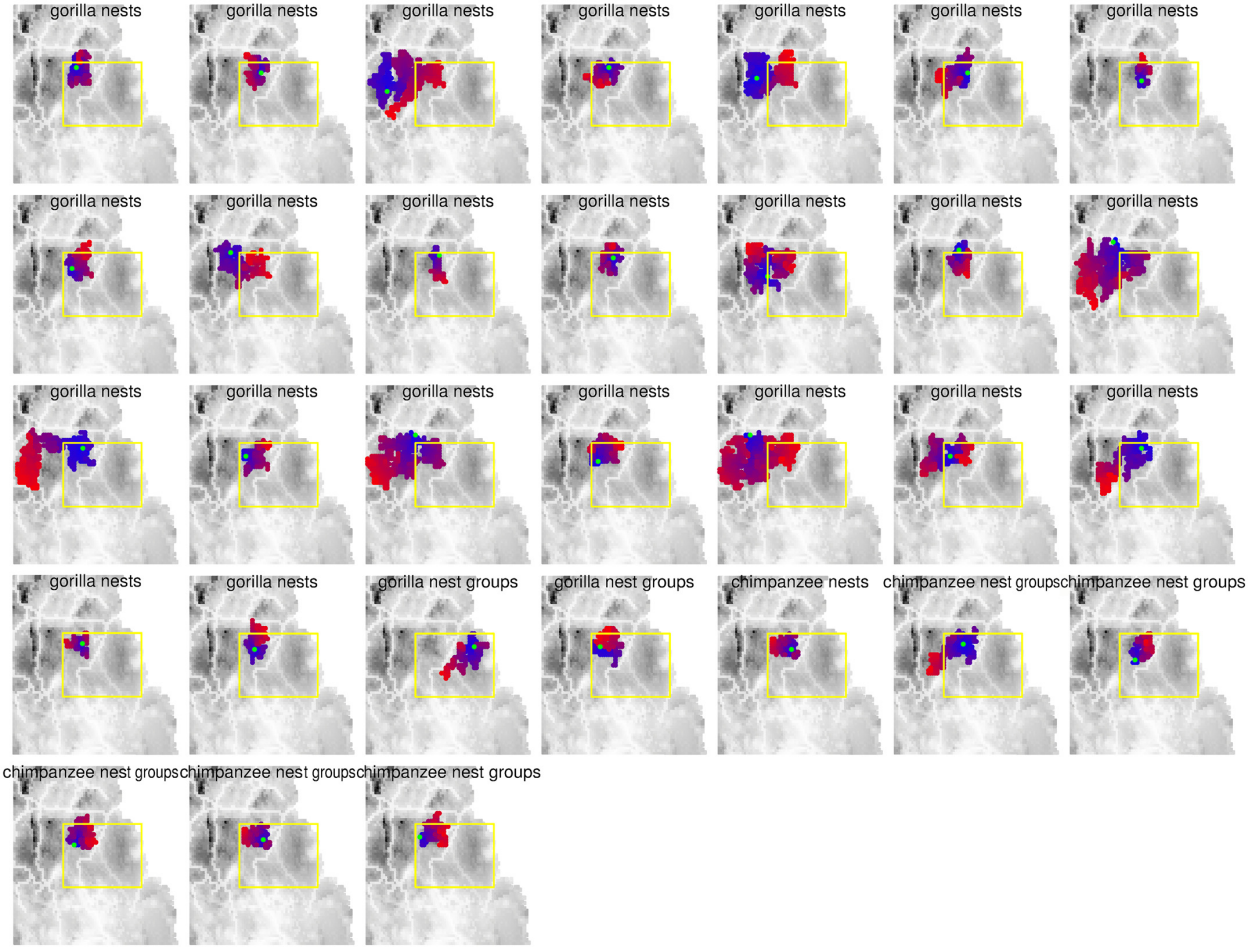
Simulated outbreak patterns for those models in the 95% best model confidence sets for *G*. *g*. *gorilla* nests and nest groups (Fig 3a and 3b, respectively) as well as for *P*. *t*. *troglodytes* nests and nest groups (Fig 3c and 3d, respectively). Each map shows the development of one simulated outbreak in time and space. The green cell depicts the origin of the outbreak. Cells colored in blue to red depict the progression of the outbreak, with blue denoting cells infected first and red denoting cells infected last. The grey shading of the background reflects habitat suitability for great apes (with darker tones indicating more suitable habitat). Note that, with one exception (row 4, column 3), all simulated outbreaks affected a very similar part of the area where the recces were conducted (yellow rectangle).

The vast majority of the total of 26,855,656 simulations (for the four responses, namely *G*. *g*. *gorilla* and *P*. *t*. *troglodytes* nest and nest group counts) did not affect any of the cells where our recces took place. About 13% of simulated outbreaks per response reached cells where recces were conducted. However, in some of these cases, models did not converge (probably due to too few surveyed cells being affected), and hence the considered numbers of simulations that affected the surveyed area were 725,920 (*G*. *g*. *gorilla* nests), 730,817 (*G*. *g*. *gorilla* nest groups), 726,062 (*P*. *t*. *troglodytes* nests) and 732,249 (*P*. *t*. *troglodytes* nest groups). Each of the four best model confidence sets, based on a cumulative AIC model weight of 0.95, comprised only a very small fraction of the respective total number of models considered (*G*. *g*. *gorilla* nests: 329; *G*. *g*. *gorilla* nest groups: 34; *P*. *t*. *troglodytes* nests: 17; *P*. *t*. *troglodytes* nest groups: 82) (Table 1). A simulated outbreak at a given location frequently led to several models with the exact same AIC because different outbreak dates and/or simulations led to the same pattern with regard to which of the surveyed cells were affected at the time when the surveys took place. Accordingly, the numbers of simulated outbreaks that appeared in the four best model confidence sets were only 23 (*G*. *g*. *gorilla* nests), 2 (*G*. *g*. *gorilla* nest groups), 1 (*P*. *t*. *troglodytes* nests) and 5 (*P*. *t*. *troglodytes* nest groups) (Fig 4). With one exception (Fig 4b, left), all simulated outbreaks in the four best model confidence sets led to similar patterns, in that the northwestern corner of the study area was affected.

**Table 1:** Summary results of simulations for the four responses.

Species	Response	Total no. models	No. models in conf. set	Model	AIC	dAIC	wAIC
*G*. *g*. *gorilla*	nests	6711072	329	Best	13429	0.0	0.10021
				Worst in conf.-set	13441	12.4	0.00020
				Null	13500	71.8	<0.00001
	nest groups	6715969	34	Best	7683	0.0	0.05447
				Worst in conf.-set	7690	6.9	0.00174
				Null	7742	59.1	<0.00001
*P*. *t*. *troglodytes*	nests	6711214	17	Best	10429	0.0	0.05820
				Worst in conf.-set	10429	0.0	0.05820
				Null	10545	116.0	<0.00001
	nest groups	6717401	82	Best	7246	0.0	0.02673
				Worst in conf.-set	7253	7.3	0.00068
				Null	7339	93.0	<0.00001

Shown are the best and the worst model in the confidence set, AIC, delta AIC (dAIC), AIC weight (wAIC) for the best and worst model in the 95% best model confidence set (models up to a cumulative AIC weight of 0.95); the total number of models evaluated and number of models in the 0.95 confidence set.

**Fig 5:**
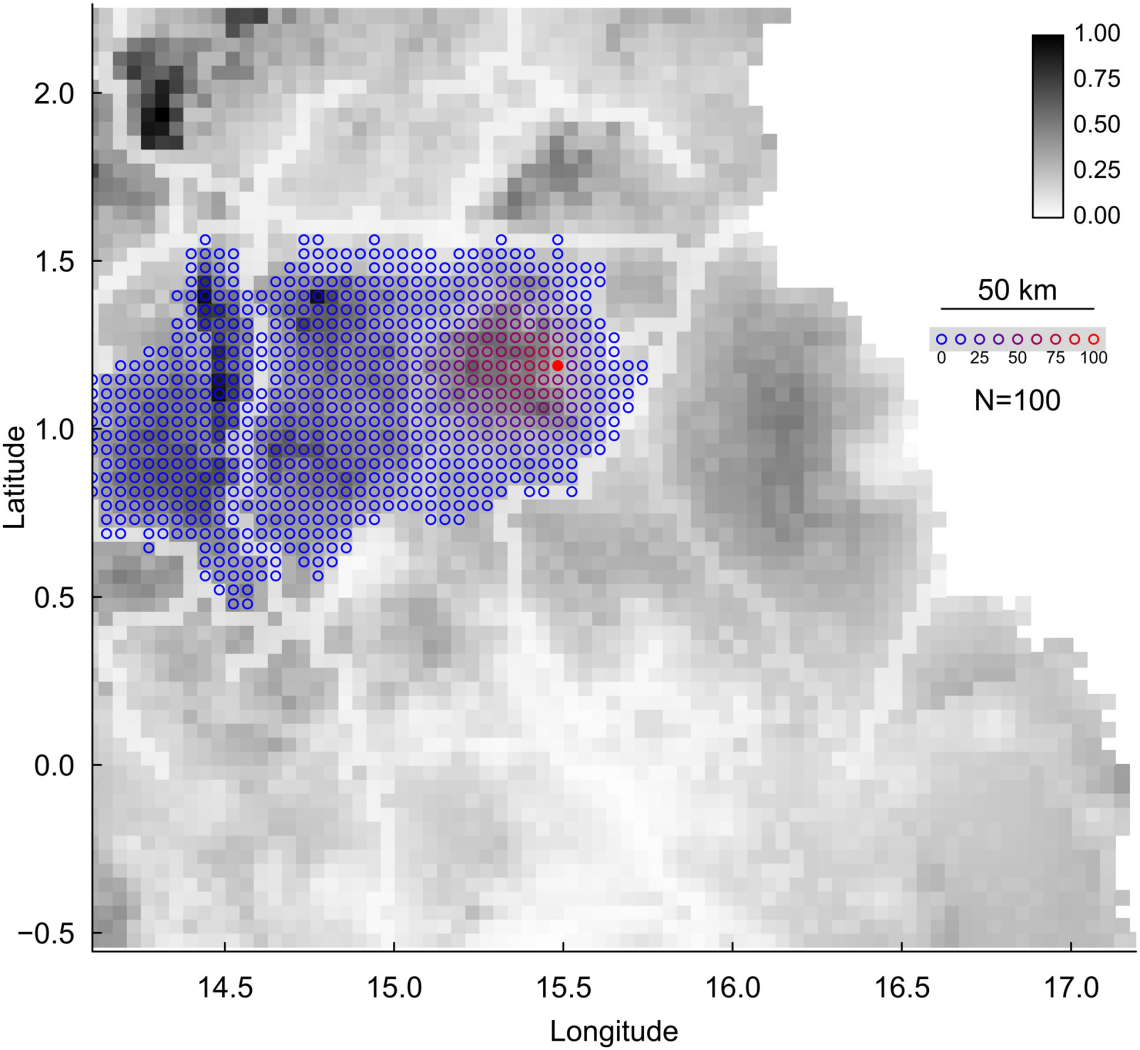
Proportions of simulated outbreaks from a given cell (filled red) affecting other cells in its vicinity. Cells affected at least once are depicted by colored circles whereby the redder the circle, the larger the proportion of simulations leading to the respective cell eventually being affected. Note that a large proportion of the cells were affected by only a small proportion of the simulations. Also note that in none of the simulations did the epidemic cross the NR2 road heading from northeast through the center of the area. The grey shading of the background reflects habitat suitability for great apes (with darker tones indicating more suitable habitat).

Most simulations affected only a small number of cells, although occasionally individual outbreaks affected considerable proportions of the study area. There was substantial variation between different simulations starting from the same point of origin. In fact, repeated simulations occasionally led to strikingly different patterns, and frequently any given cell was only rarely affected by an outbreak from a given location, particularly if it was further away from the location where the outbreak was simulated. Furthermore, many of the simulated disease spreads in the landscape halted at roads or rivers, both of which are surrounded by reduced ape habitat suitability values (Fig 5).\

## Discussion

Through our statistical model, we have attempted to link epidemiological theory with empirical data. Results presented here highlight three main findings: (*i*) although there was considerable variation in the pattern of simulated disease spread, the best models predicted emergence at locales in the western region of OKNP; (*ii*) simulations of disease emergence and spread produced a very small number of hypothetical impact profiles that matched empirical data from our ape surveys in an affected region of the study area; and (*iii*) the combination of empirical data on ape nesting patterns and ape habitat suitability indicated roads were consistently associated with attenuation of modeled disease spread in ape populations.

The parameters of our model were based on ape suitable habitat and a suite of associated environmental variables. The model assumed disease spread was density dependent and used suitable environmental conditions as a proxy for great ape density. The results of the model predict emergence of the presumed disease outbreak outside the range of our observed ape populations. Low discrepancy between predicted emergence locales and known outbreaks suggest there may well be identifiable environmental factors underpinning this phenomenon. The results of the model showed good broad visual agreement with predictive maps of where large-scale ape mortality is believed to have occurred prior to 2005, based on available ape nest data [3,4,5,6].

Locales of high risk of potential emergence corresponded primarily to highly suitable ape habitat characterized by low topographic relief and mixed species forest with relatively open canopy [18]. This apparent host density dependence is consistent with an infectious disease etiology. However, outputs of our model did not indicate the eastern portion of the study region (east of OKNP) to be a likely location of disease emergence, despite apparently high ape densities there.

The vast majority of cells west of NR2 show very low numbers of apes nests and nest groups (Fig 3). These low numbers are unlikely to be due to movement of apes from those cells to adjacent cells. Western lowland gorillas typically remain within home ranges and ranging movements are usually dictated by food availability. Gorilla home ranges in the Lossi Gorilla Sanctuary–a region of similar food availability to our study area–average only 11km^2^ [19]. We note that the use of recces to monitor ape abundance was a study limitation. We used recces because of our interest in expediently identifying great ape carcasses (transects are more time-consuming and expensive), however, the regional consistency of trends over the surveillance period suggests we observed real changes in population density.

Natural barriers within the landscape may also influence transmission dynamics [20] and major rivers in Central Africa have been shown to play an important role in directing gene flow in *G*. *g*. *gorilla* [21]. Our post-epizootic nest surveys also found higher numbers of *G*. *g*. *gorilla* and *P*. *t*. *troglodytes* nests in proximity to roads, suggesting that ape abundance in proximity to roads was low pre-epizootic and likely insufficent to facilitate disease transmission. Walsh et al. 2009 found a negative correlation with distance to roads and ape densities in the Lossi region which they interpreted as lowering the potential for disease transmission [13]. Understanding the tradeoffs of negative and positive road impacts on conservation and disease spread will need further study.

The spatial and temporal dynamics of this unusual mortality event are most consistent with an infectious etiology. Without confirmatory diagnostic testing, the specific pathogen(s) involved in the 2006–2007 outbreak cannot be determined. A number of pathogens have been associated with wild Africa ape mortality, including anthrax in West and Central Africa [22,23], human paramyxoviruses in West Africa [24], *Streptococcus pneumoniae* and *Pasteurella multocida* in West Africa [25] and ebolaviruses in West and Central Africa [3,6,26,27]. Of these, only ebolavirus has been associated with widespread ape mortality in greater Gabon and Congo region [3,5,6,7,27]. The results of our model represent an important step in understanding variables related to disease ecology in Central African apes and may help guide future intervention strategies, such as vaccination of free-ranging apes against select pathogens. The results also highlight the need for more surveillance and research to improve conservation and public health efforts in the region.

The northern Congo region is home to the majority of remaining western lowland gorillas and cenrtal chimpanzees, making it region a priority for their protection and long-term maintenance [28,29], as well as for research and tourism. As disease is amongst the leading threats to these species [1], it is imperative to gain a better understanding of disease transmission dynamics in great apes, including potential reservoirs and vectors. One aspect that deserves additional scrutiny is the differential impact of various disease processes on gorillas and chimpanzees, given their different ecologies and social dynamics.

Continuing regular large-scale surveys throughout this ape-rich region will be important in order to monitor ape abundance and detect, at a minimum, drastic populations declines. Any surveys should not overlook such factors as human disturbance, particularly in relation to hunting pressure, roads and logging operations. There is a need for industrial logging companies to continue to work in collaboration with conservation organizations, as logging personnel working in remote areas are both likely to detect, and potentially be impacted by, wildlife disease outbreaks. Continued eduction of and surveillance by local communities (e.g. hunters) is imperative, as they are at once the most likely demographic to detect wildlife mortality in real time and most at risk of zoonotic disease transmission.

## Supporting Information

S1 Table List of covariates used. (DOCX)

S2 Table Results of the Factor Analysis. (DOCX)
