# Supplementary material for: Spatial and Temporal Dynamics of a Mortality Event among Central African Great Apes
Source: PLoS One. 2016 May 18;11(5):e0154505. doi: 10.1371/journal.pone.0154505 (PMC4871434; doi:10.1371/journal.pone.0154505)
Supplement: S1 Table — (DOCX) [file pone.0154505.s001.docx]

**S1 Table. List of covariates used**

| **Covariate** | **Details** |
| --- | --- |
| prec_seas | BIO15 = Precipitation Seasonality (Coefficient of Variation)^e^ |
| tr.prec_wet_mo^a^ | BIO13 = Precipitation of Wettest Month^e^ |
| prec_dry_mo | BIO14 = Precipitation of Driest Month^e^ |
| tr.prec_dry_qua^b^ | BIO17 = Precipitation of Driest Quarter^e^ |
| tr.prec_co_qua^b^ | BIO19 = Precipitation of Coldest Quarter^e^ |
| prec_wet_qua | BIO16 = Precipitation of Wettest Quarter^e^ |
| tr.hhi^a^ | Human influence index^j^ |
| temp_seas | BIO4 = Temperature Seasonality^e^ |
| prec_wa_qua | BIO18 = Precipitation of Warmest Quarter^e^ |
| annual_temp | BIO1 = Annual Mean Temperature^e^ |
| temp_wa_qua | BIO10 = Mean Temperature of Warmest Quarter^e^ |
| max_temp | BIO5 = Max Temperature of Warmest Month^e^ |
| temp_wet_qua | BIO8 = Mean Temperature of Wettest Quarter^e^ |
| temp_co_qua | BIO11 = Mean Temperature of Coldest Quarter^e^ |
| min_temp | BIO6 = Min Temperature of Coldest Month^e^ |
| temp_dry_qua | BIO9 = Mean Temperature of Driest Quarter^e^ |
| temp_ra_day | BIO2 = Mean Diurnal Temperature Range^e^ |
| Forest | Globecover 2009, class 40^f^ |
| Mosaic | Globecover 2009, class 30^f^ |
| Isotherm | BIO3 = Isothermality^e^ |
| an_temp_rang | BIO7 = Temperature Annual Range^e^ |
| tr.dist.road^c^ | Closest distance to road^g^ |
| tr.hum_dens^c^ | Human population density^h^ |
| ann_prec | BIO12 = Annual Precipitation^e^ |
| tr.dist.riv^c^ | Closest distance to river^g^ |
| tr.cti^d^ | Compound topographic index^i^ |

^a^transformed as x'=sqrt(x-min(x))

^b^transformed as x'=max(sqrt(max(x)-x))-sqrt(max(x)-x)

^c^transformed as x'=sqrt(x)

^d^transformed as x'=log(x)

^e^Hijmans RJ, Cameron SE, Parra JL, Jones PG and Jarvis A. Very high resolution interpolated climate surfaces for global land areas. International Journal of Climatology. 2005;25: 1965–1978.

^f^Arino O, Ramos Perez JJ, Kalogirou V, Bontemps S, Defourny P and Van Bogaert E. Global land cover map for 2009 (GlobCover 2009). 2012.

^g^Defense Mapping Agency. Development of the Digital Chart of the World: U.S. Government Printing Office, Washington, D.C. 1992.

^h^Center for International Earth Science Information Network (CIESIN), Columbia University and Centro Internacional de Agricultura Tropical (CIAT). 2005.

^i^Hydro 1k dataset, [Data available from the U.S. Geological Survey.](http://eros.usgs.gov/#/About_Us/Customer_Service/Data_Citation)

^j^Fotheringham AS. Spatial structure and distance decay parameters. Annals of the Association of American Geographers. 1981;71: 425–436.
