# Supplementary material for: Spatial and Temporal Dynamics of a Mortality Event among Central African Great Apes
Source: PLoS One. 2016 May 18;11(5):e0154505. doi: 10.1371/journal.pone.0154505 (PMC4871434; doi:10.1371/journal.pone.0154505)
Supplement: S2 Table — (DOCX) [file pone.0154505.s002.docx]

**S2 Table. Results of the Factor Analysis.** Indicated are the loadings of the original covariates on the factors derived (largest absolute loading per covariate indicated in bold), the Eigenvalues of the factors and the percentage of the total variance they explained.

| **Covariate** | **factor 1** | **factor 2** | **factor 3** | **factor 4** | **factor 5** | **factor 6** |
| --- | --- | --- | --- | --- | --- | --- |
| prec_seas | **-0.97** | -0.03 | -0.03 | 0.03 | 0.09 | 0.04 |
| tr.prec_wet_mo | **-0.96** | -0.21 | 0.00 | 0.06 | 0.02 | 0.01 |
| prec_dry_mo | **0.95** | -0.15 | -0.06 | -0.20 | -0.03 | 0.04 |
| tr.prec_dry_qua | **0.93** | -0.15 | 0.00 | -0.18 | -0.05 | 0.05 |
| tr.prec_co_qua | **0.93** | -0.05 | 0.01 | -0.12 | -0.02 | -0.01 |
| prec_wet_qua | **-0.92** | -0.22 | 0.06 | 0.18 | 0.18 | -0.06 |
| tr.hhi | **0.91** | 0.26 | 0.10 | 0.04 | 0.16 | -0.06 |
| temp_seas | **-0.84** | 0.10 | -0.07 | -0.26 | -0.07 | 0.06 |
| prec_wa_qua | **-0.68** | 0.01 | 0.07 | 0.14 | 0.02 | -0.21 |
| annual_temp | 0.01 | **0.99** | -0.01 | 0.01 | -0.02 | 0.05 |
| temp_wa_qua | -0.05 | **0.99** | -0.01 | -0.03 | -0.03 | 0.05 |
| max_temp | -0.01 | **0.99** | 0.00 | -0.05 | -0.10 | 0.07 |
| temp_wet_qua | -0.04 | **0.99** | -0.02 | -0.04 | -0.02 | 0.06 |
| temp_co_qua | 0.21 | **0.97** | 0.01 | 0.00 | -0.01 | 0.03 |
| min_temp | -0.25 | **0.93** | 0.04 | 0.24 | 0.00 | 0.02 |
| temp_dry_qua | 0.44 | **0.85** | 0.01 | -0.07 | 0.03 | 0.02 |
| temp_ra_day | 0.10 | **-0.67** | -0.15 | -0.17 | -0.53 | 0.21 |
| Forest | 0.01 | -0.02 | **-0.99** | -0.04 | -0.06 | 0.05 |
| Mosaic | 0.01 | 0.01 | **0.99** | 0.03 | 0.07 | -0.06 |
| Isotherm | -0.58 | -0.11 | -0.01 | **0.71** | -0.12 | 0.02 |
| an_temp_rang | 0.59 | -0.25 | -0.11 | **-0.70** | -0.20 | 0.09 |
| tr.dist.road | 0.12 | -0.01 | 0.10 | -0.02 | **0.68** | 0.22 |
| tr.hum_dens | 0.40 | 0.03 | 0.16 | 0.47 | **-0.58** | 0.12 |
| ann_prec | -0.30 | -0.48 | 0.19 | 0.27 | **0.56** | -0.30 |
| tr.dist.riv | 0.19 | -0.12 | 0.08 | 0.07 | 0.07 | **-0.74** |
| tr.cti | 0.27 | 0.03 | -0.01 | 0.09 | 0.19 | **0.73** |
| Eigenvalue | 8.68 | 7.42 | 2.09 | 1.61 | 1.57 | 1.36 |
| % var. explained | 33.40 | 28.52 | 8.02 | 6.19 | 6.03 | 5.22 |
